# Physical Activity Is Associated with Higher Retinal Microvascular Density in Uveal Melanoma and Nevus

**DOI:** 10.3390/cancers18152502

**Published:** 2026-08-04

**Authors:** Theresa Walz, Freerk T. Baumann, Katharina Leuchte, Damir Zubac, Philomena Wawer Matos Reimer, Konrad R. Koch, Michael Mendes Wefelnberg

**Affiliations:** 1 Department of Pediatric Hematology and Oncology, University Hospital Bonn, 53127 Bonn, Germany; theresa.walz@ukbonn.de; 2 Department of Internal Medicine I, Center for Integrated Oncology, University Hospital Cologne, 50937 Cologne, Germanydamir.zubac@uk-koeln.de (D.Z.);; 3 National Center for Cancer Immune Therapy (CCIT-DK), Department of Oncology, Copenhagen University Hospital Herlev, 2730 Herlev, Denmark; 4 Department of Ophthalmology, Faculty of Medicine and University Hospital Cologne, University of Cologne, 50937 Cologne, Germanykonrad.koch@uk-koeln.de (K.R.K.)

**Keywords:** physical activity, uveal melanoma, optical coherence tomography angiography, tumor micro-environment, microvascular density

## Abstract

**Background/Objectives**: The aim of the present study was to assess the influence of physical activity on retinal microvascularization in patients with uveal melanoma (UM) and nevus. In addition, vascular differences between tumor and nevus patients as well as the interaction effect of physical activity (PA) and group on retinal microvascular density (MVD) were analyzed. **Methods**: This cross-sectional, observational clinical study was conducted at the outpatient ophthalmic oncology department at the Center for Integrated Oncology of the University Hospital Cologne (between April 2024 and July 2025). Patients with confirmed uveal melanoma or choroidal nevus were enrolled. Physical activity and retinal MVD via optical coherence tomography angiography were assessed. In addition, regression analyses were performed. **Results**: A total of 42 participants were enrolled with a mean age of 56.14 (±13.48) years (nevus) and 63.45 (±13.76) years (UM). UM eyes (*N* = 20) demonstrated significantly reduced MVD in both the superficial and deep layer compared to Nevus (*N* = 22, all *p* < 0.001, η^2^ = 0.155–0.190), while UM eyes showed greater mean vessel length (B = 5.54, *p* = 0.009, η^2^ = 0.104). Physical activity was positively associated with MVD in the superficial layer, including vessel area density (B = 1.07, *p* = 0.022, η^2^ = 0.133) and vessel length density (B = 0.28, *p* = 0.028, η^2^ = 0.119), independent of group. No significant associations were found in the deep layer and no interaction effects between group and PA were detected. **Conclusions**: Our findings suggest that habitual PA is positively associated with retinal MVD in the superficial layer, independent of diagnostic group, and may partially counteract tumor-induced as well as treatment-related microvascular disruption. As a low-risk and accessible intervention, PA holds promise as a supportive strategy in the oncological management of UM.

## Simple Summary

Uveal melanoma is a rare but aggressive eye tumor associated with high metastatic risk. Physical activity is increasingly recognized as a beneficial supportive measure in cancer care, yet its potential effects on the blood vessel environment in and around eye tumors remain unexplored. Since the microvasculature of the retina can be visualized non-invasively using modern imaging technology, the eye offers a unique opportunity to study vascular changes associated with physical activity in cancer patients. In this study, we investigated whether habitual physical activity is positively associated with retinal microvascular density in patients with uveal melanoma or benign eye lesions. Higher physical activity levels were associated with greater microvascular density, independent of diagnosis. These findings suggest that regular physical activity may help preserve vascular health in the eye and could represent a simple, accessible, and low-risk supportive strategy for patients with uveal melanoma. Future prospective interventional studies should investigate whether such effects could also translate into increased treatment effectiveness or mitigation of radiation retinopathy.

## 1. Introduction

Uveal melanoma (UM) is the most common primary intraocular malignancy in adults and is characterized by a high mortality rate [1]. Among solid malignancies, UM exhibits a particularly pronounced hypoxic tumor microenvironment (TME) [2], which plays a central role in promoting cancer progression, metastatic dissemination, immune evasion, and resistance to therapy [3,4]. This profoundly disrupted TME is reflected in measurable alterations of the retinal microvasculature, including decreased microvascular density (MVD) [5].

In addition to the effects of the tumor itself, radiotherapy, most commonly brachytherapy, the standard treatment for UM, is associated with treatment-related damage to the retinal microvasculature. This includes radiation retinopathy which is characterized by progressive vascular damage and retinal ischemia, arising from radiation-induced structural and functional alterations of the retinal microvasculature [6,7].

Exercise and PA have the potential to benefit retinal health in several ways [8]. Exercise can elevate ocular perfusion pressure by up to 190%, leading to an increase in both choroidal and retinal blood flow [9]. Those changes in ocular blood flow are considered to have a positive impact on retinal nourishment [10].

Generally, PA and exercise show strong evidence of attenuating diverse disease- or treatment-related side effects like, e.g., fatigue, cognitive impairment, and cancer cachexia [11,12,13,14], or even of preventing recurrence [15]. Moreover, it is known that physical exercise influences tumor-specific vascular dynamics as well [16]. The potential to positively influence the TME through PA by restructuring the tumor’s vascular system is much discussed. This may improve tumor perfusion, reduce hypoxia, inhibit tumor growth, and improve the effectiveness of radiotherapy and chemotherapy [15,17]. However, most evidence supporting these vascular effects derives from preclinical animal models, and direct in vivo demonstration of PA-induced improvements in the tumor vascular microenvironment in humans remains lacking.

In this regard, UM offers an interesting opportunity to detect PA-induced changes in the TME. The new method of optical coherence tomography angiography (OCTA) can perform fast and non-invasive measurements of ocular vessels. Recent research shows that this imaging technology even identifies PA-induced short- and long-term changes in healthy retinal microvasculature [18,19,20,21]. To date, only one case study has examined the longitudinal microvascular response to endurance exercise in patients with UM, reporting largely increased vessel elasticity following 8 weeks of high-intensity training (HIIT) [22].

While the effects of exercise on the TME have been studied in other cancer entities [16], evidence specific to habitual PA and UM remains scarce. Furthermore, as outlined above, the mechanistic evidence supporting PA-induced vascular remodeling in the TME derives predominantly from preclinical models, with lacking confirmation in human tumor tissue. The ocular localization of UM offers a unique opportunity to address this gap, as retinal microvasculature can be assessed non-invasively and with high resolution using OCTA. This allows for the quantification of potential PA-associated vascular adaptations in close proximity to the tumor. Therefore, the present study aimed to investigate whether habitual PA is associated with retinal microvascularization in patients with UM or choroidal nevus, and whether PA-related vascular effects differ between diagnostic groups.

## 2. Materials and Methods

### 2.1. Study Design

This observational study was conducted as part of the EyeCanMoveIt (ECMI) project at the Center for Integrated Oncology (CIO) at University Hospital Cologne. The study was conducted in accordance with the Declaration of Helsinki, approved by the Ethics Committee of the Medical Faculty of the University of Cologne (approval number: 22-1062), and registered in the German Clinical Trials Register (DRKS-ID: DRKS00031207; registration date: [3 February 2023]). The consecutive patients were recruited at the outpatient ophthalmic-oncological department at the CIO. The data collection period was from April 2024 to July 2025. This study is reported in accordance with the STROBE guidelines for observational studies (Table S1).

**Figure 1:**
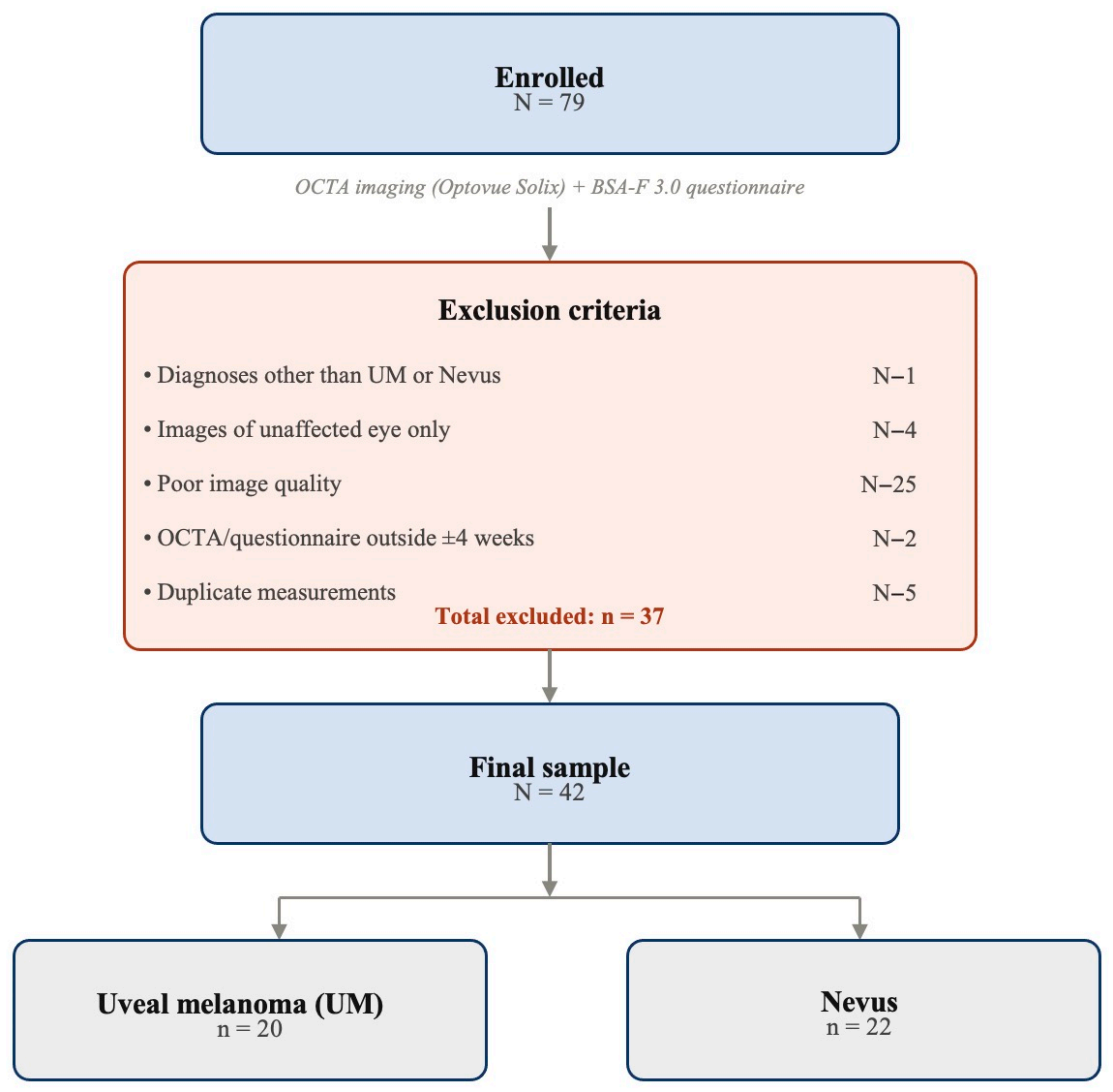
Participant flow diagram. Of 79 enrolled patients, 37 were excluded, resulting in a final sample of 42 patients (20 UM, 22 Nevus).

PA was assessed using a modified version of the BSA-F 3.0 (Bewegungs- und Sportaktivität Fragebogen) [23], completed on the day of ophthalmologic examination. OCTA images of the retina of the affected eye were then taken with a commercial spectral domain OCTA-system (Optovue Solix, Visionix, Jerusalem, Israel). The superficial and deep layer were used as the primary examination layers. A detailed overview of patient recruitment and inclusion is provided in Figure 1.

### 2.2. Study Population

Due to the explorative nature of this investigation, no formal power calculation was performed. The intended sample size was *N* = 40, with equal allocation across both groups (*n* = 20 with UM and *n* = 20 with choroidal nevus), determined by the availability of suitable participants during the recruitment period. Prerequisite for study inclusion were (1) a diagnosis of choroidal melanoma or choroidal nevus, (2) images of the retinal ocular vessels of the affected eye via OCTA, (3) an image quality score of at least 5–7 (QS ≥ 7 for healthy eyes and QS ≥ 5 in the tumor-afflicted eye with visible tumor in the image) out of 10, (4) low motion artefact score, and (5) completion of the BSA questionnaire. Accordingly, exclusion criteria were (1) presence of other ocular disease, (2) the absence of the questionnaire data or OCTA images, (3) OCTA images of the unaffected eye and (4) poor image quality, (5) OCTA imaging and questionnaire completion not performed within the same timeframe, and (6) duplicate measurements (in case of repeated imaging, only one scan per individual was included).

### 2.3. Assessments

OCTA images were acquired using the Optovue Solix (Visionix, Jerusalem, Israel). For each patient, 6.4 × 6.4 en face images of the superficial and deep retinal layers of the affected eye were obtained (OS or OD, respectively). Image analysis was performed using OCTAVA software. Filter settings were standardized based on Untracht et al. [24]. Frangi filter with kernel size four was applied for pre-processing, no median filter was used, and Fuzzy thresholding was selected for segmentation. Twig size was set to the default value of eight. Considering the biological relevance of the metrics and the assessments described by Untracht et al. [24], the following OCTA parameters served as dependent variables: nodes, vessel area density (VAD), total vessel length, vessel length density_pct (VLD_pct), vessel length density_mm (VLD_mm), mean length, mean diameter, branchpoint density (BD), and mean tortuosity. Fractal dimension was excluded due to absence of variance across the study sample.

Weekly PA was assessed using the BSA-F 3.0 questionnaire (Bewegungs- und Sportaktivität Fragebogen), a validated self-completion instrument based on the FITT framework, capturing frequency, duration, and type of activity [23]. PA was operationalized as the total weekly duration (min/week) of leisure-time movement and sports activity, in line with the BSA-F scoring procedure, and subsequently converted into metabolic equivalent of task minutes per week (MET-min/week) to enable standardized quantification of overall activity load (for details see Section 2.4).

### 2.4. Statistical Analysis

Statistical analyses were performed using jamovi (version 2.3) and graphical visualizations were generated using GraphPad Prism 11 (GraphPad Software, San Diego, CA, USA). Continuous demographic and clinical characteristics are presented as mean (M) ± standard deviation (SD), and categorical variables are reported as frequencies and percentages. To evaluate the impact of disease group (UM eyes vs. nevus controls) and physical activity levels on superficial macular microcirculation parameters, multiple linear regression analyses (ANCOVA framework) were conducted. Separate models were fitted for nine primary OCTA metrics for the superficial vascular plexus (SVP) and deep vascular plexus (DVP) extracted via the OCTAVA software.

All models were adjusted for the predefined confounding covariates of age (at study inclusion) and the Charlson Comorbidity Index (CCI). Physical activity was entered into the models as a log10-transformed MET-min/week continuous predictor (log10_MET) to account for the highly skewed distribution of the raw metabolic equivalents.

The ordinary least-squares (OLS) method was utilized for parameter estimation. Prior to hypothesis testing, the statistical assumptions underlying multiple linear regression were systematically verified for each model. Multicollinearity among predictors was evaluated using Tolerance and Variance Inflation Factors (VIF). The independence of residuals was assessed via the Durbin–Watson statistic. Assumptions of linearity, homoscedasticity, and the normality of residuals were evaluated through visual inspection of standardized residual scatterplots and normal probability (P-P) plots, supplemented by Shapiro–Wilk tests. Before final model selection, the presence of an interaction effect between disease group and physical activity level was evaluated. Overall model fit was assessed using the coefficient of determination (R^2^). To determine the magnitude and clinical relevance of the independent effects, Eta-squared (η^2^) values were calculated as measures of effect size and interpreted according to Cohen’s guidelines [25]. Statistical significance was set a priori at α = 0.05 using two-sided tests. Given the number of models tested (nine OCTA parameters across two vascular plexus layers), no correction for multiple comparisons was applied. Associations involving PA should therefore be interpreted as exploratory and hypothesis-generating rather than confirmatory.

### 2.5. Ethical Considerations

The study protocol, patient information, and informed consent form were submitted to and approved by the Ethics Committee of the Medical Faculty of the University of Cologne (approval number: 22-1062). The study was conducted in accordance with the Declaration of Helsinki. Written informed consent was obtained from all participants prior to study inclusion.

## 3. Results

### 3.1. Study Population

**Figure 2:**
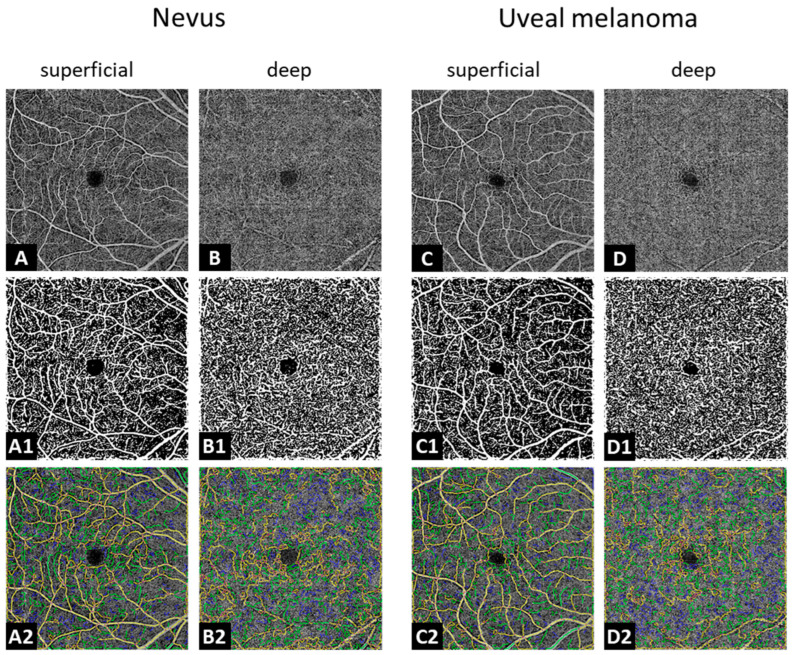
Original and processed (by OCTAVA) OCTA images of the retina in SVP and DVP in patients with nevus and UM. (**A**) Nevus, SVP; (**A1**) Nevus, skeletonized image, SVP; (**A2**) Nevus, angio-overlay, SVP; (**B**) Nevus, DVP; (**B1**) Nevus skeletonized image, DVP; (**B2**) Nevus angio-overlay, DVP; (**C**) UM, SVP; (**C1**) UM, skeletonized image, SVP; (**C2**) UM, angio-overlay, SVP; (**D**) UM, DVP; (**D1**) UM, skeletonized image, DVP; (**D2**) UM, angio-overlay, DVP.

The final sample comprised 42 participants (UM: *N* = 20, nevus: *N* = 22). The mean age was 56.14 ± 13.48 years for nevus and 63.45 ± 13.76 years for UM (*p* = 0.036). The two groups did not differ significantly in sex, height, weight, BMI or CCI (all *p* > 0.05), but differed significantly in days between diagnosis and assessment (*p* = 0.006), lesion size and thickness (both *p* > 0.05). Detailed characteristics are provided in Table 1. Representative OCTA images of the SVP for both diagnostic groups are displayed in Figure 2.

### 3.2. Association Between Physical Activity and Retinal Microvasculature Across Groups

Multiple linear regression analyses were conducted for nine OCTA parameters in both the SVP and DVP. Results are summarized in Table 2. Assumption diagnostics were evaluated across all 18 models to ensure statistical validity. Multicollinearity was within acceptable reference parameters. Across all models, Variance Inflation Factors (VIF) fell well below the critical threshold of 5.0 (ranging from 1.2 to 3.3), and Tolerance values remained safely above 0.20 (ranging from 0.31 to 0.87). The assumption of independent errors was met, as Durbin–Watson statistics clustered closely around the ideal value of 2.0, ranging from 1.68 to 2.13. Visual inspections of standardized residual scatterplots confirmed the assumptions of linearity and homoscedasticity, displaying a random, even distribution of errors across all models. Finally, normal P-P plots and Shapiro–Wilk tests confirmed that the residuals approximated a normal distribution (*p* > 0.05), indicating no severe deviations from normality.

In brief, group (UM vs. Nevus) was a significant predictor for most parameters, including nodes, VAD, total vessel length, VLD (%), VLD (mm^−1^), and BD in both plexus layers (all *p* < 0.05), with UM showing lower values. Mean length was also significantly associated with group in both plexus layers, with UM showing higher values compared to Nevus (SVP: *p* = 0.009, η^2^ = 0.104; DVP: *p* = 0.003, η^2^ = 0.190). Mean diameter and mean tortuosity did not differ significantly between groups.

**Figure 3:**
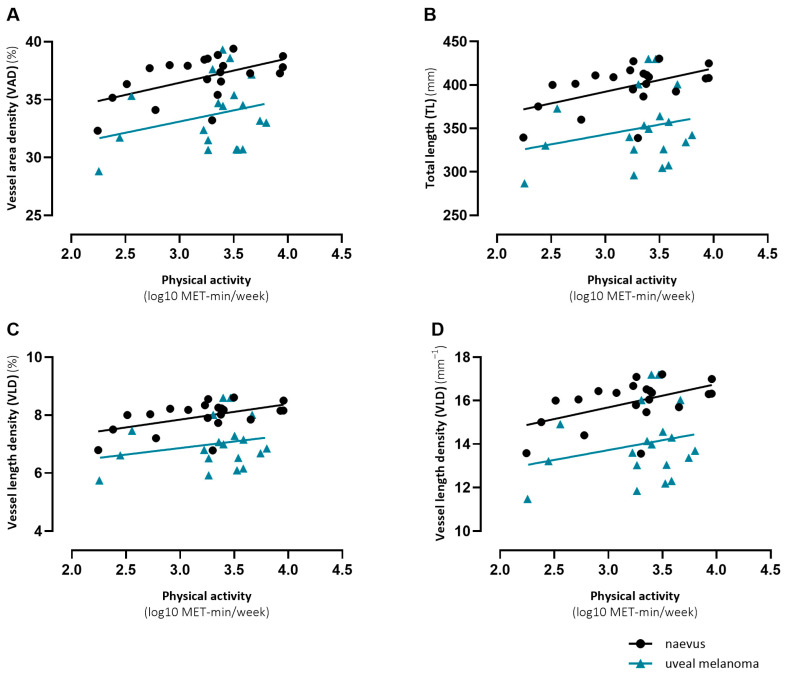
Association between physical activity and superficial macular microcirculation. Adjusted parallel regression lines and data points for the nevus group (circles) and UM group (triangles) across four significant superficial OCTA parameters (*N* = 42): (**A**) vessel area density (VAD, %) [Model R^2^ = 0.536]; (**B**) total vessel length (TVL, mm) [Model R^2^ = 0.563]; (**C**) vessel length density (VLD, %) [Model R^2^ = 0.563]; and (**D**) vessel length density mm (VLD, mm^−1^) [Model R^2^ = 0.563]. Lines are fitted using an ordinary least-squares shared-slope model adjusted for age and Charlson Comorbidity Index (CCI). All parameters show a highly significant main effect for disease group, with marked microvascular reduction in UM eyes (*p* < 0.001). Concurrently, a significant positive main effect for physical activity (log10_MET-min/week) is confirmed by the uniform upward slopes across all models (VAD: B = 1.07, *p* = 0.022, η^2^ = 0.133; TVL: B = 13.98, *p* = 0.028, η^2^ = 0.120; VLD_pct: B = 0.28, *p* = 0.028, η^2^ = 0.119; VLD_mm: B = 0.56, *p* = 0.028, η^2^ = 0.120).

Regarding PA, log10-transformed MET-min/week (log10_MET) was significantly associated with VAD, total vessel length, VLD (%), and VLD (mm^−1^) (all *p* < 0.05, η^2^ = 0.119–0.133) in the SVP, with higher PA levels corresponding to higher values (Figure 3). No significant associations were observed for nodes, mean length, mean diameter, BD, or mean tortuosity. In the DVP, no significant associations between log10_MET and any OCTA parameter were observed. No significant interaction effects between group and PA were detected for any parameter in either plexus layer.

## 4. Discussion

The present study investigated PA-associated alterations in retinal microvasculature in UM and nevus patients. Our results reveal that higher PA levels are positively associated with increased MVD in the SVP for both UM and nevus patients—suggesting that exercise may help restore microvascular function, even in retinal tissue severely compromised by tumor and radiation.

The underlying mechanisms may include PA-induced increases in microvascular shear stress, which stimulate eNOS activity and endothelial NO release, thereby promoting vasodilation, capillary remodeling, angiogenic signaling and antioncogenic effects [26,27,28,29,30]. Although these mechanisms have primarily been described in other tumor entities, they may similarly apply to the ocular TME in UM.

Although significant differences between diagnostic groups were detected across most microvascular parameters in both layers, there was no significant interaction effect detected between PA and group. The observed group differences in retinal microvascular parameters are consistent with previous findings in the literature. In fact, it is reported that density-related parameters are significantly decreased in UM-affected eyes compared to non-tumor eyes [5,31,32], which is in line with the present results showing significantly lower VAD, VLD (%), and VLD (mm^−1^) in UM patients. The consistency of the present findings with the existing literature not only supports the validity of the OCTA measurements employed in this study but also suggests that the observed microvascular differences reflect genuine tumor-induced remodeling rather than methodological artifacts. The TME is characterized by pathologic angiogenesis, which frequently results in a chaotic, inefficient, and hypoxic vascular architecture associated with reduced effective perfusion [33,34]. The observed reduction in MVD in UM eyes is thus consistent with a TME dominated by leaky, poorly perfused vessels that are difficult to detect by OCTA, as the rapid vascular expansion associated with the angiogenic switch may displace or replace functional capillary networks [35,36]. Furthermore, treatment-related damage to the retinal microvasculature represents an additional severe concern, as radiotherapy in UM has been linked to vascular damage, reduced vessel density, and thus, impaired perfusion [37,38].

Against this background of compounding tumor- and radiation-induced microvascular disruption, the present findings suggest that PA may counteract these effects. Higher PA levels were positively associated with retinal MVD in the SVP, with PA explaining approximately 12–16% of the variance in microvascular density parameters (η^2^ = 0.119–0.133), while the full models, including diagnostic group, explained up to 54–56% of the total variance (R^2^ = 0.54–0.56). These effect sizes are classified as medium to large according to Cohen’s guidelines [25], which is noteworthy for an observational study in which numerous uncontrolled factors inherently contribute to microvascular variability. It should be noted that VAD, total vessel length, VLD (%), and VLD (mm^−1^) are closely related, largely collinear indicators of vessel density. The associations reported here should therefore not be interpreted as four independent lines of evidence, but rather as reflecting a single underlying association with overall MVD in the SVP. This indicates that PA-induced vascular adaptations may help restore or improve microvascular function. Notably, no significant interaction effects between group and PA were detected for any of the OCTA parameters, suggesting that the influence of PA on retinal microvasculature is independent of the underlying diagnosis. Consequently, PA appears to exert a reorganizing effect on the retinal microvasculature in both healthy and tumor-affected eyes. The observed increase in MVD associated with higher PA levels may therefore reflect enhanced vascular integrity and improved capillary perfusion. This interpretation is further supported by the technical properties of OCTA. Since the method detects vessels based on motion contrast, well-perfused vessels are more reliably visualized, whereas those with slow or absent flow due to insufficient perfusion may be underestimated [35,36].

Additionally, it should be noted that 75% (*n* = 15) of the UM patients in the present study had priorly received radiotherapy, with a mean time since treatment of approximately 23 months (see Table 1), compounding the tumor-induced microvascular disruption with treatment-related vascular damage. This further underscores the potential clinical relevance of PA-induced vascular adaptations in this population, as they may help attenuate adverse effects arising from both the tumor itself and its treatment.

Notably, the positive association between PA and MVD emerged exclusively within the SVP, with no significant effects observed in the DVP. This may be explained by structural and functional differences between the two vascular plexus layers. The SVP contains larger arterioles and venules, whereas the DVP predominantly consists of densely interconnected and metabolically active capillary networks [35,39,40]. Consequently, vascular density-related adaptations, such as changes in VAD and VLD, may be more readily detectable in the SVP, while the DVP may be more susceptible to transient alterations in perfusion pressure and shear stress than to structural vascular remodeling. Furthermore, choroidal and deep retinal perfusion are thought to primarily reflect systemic hemodynamic fluctuations rather than localized vascular regulation, as the choroid exhibits only limited autoregulatory capacity compared with the retinal circulation [41,42].

The present findings may have relevant clinical implications. Increased MVD is considered a hallmark of healthy vascular aging and has been associated with greater longevity [43]. Since the eye is considered the “window to the heart” as it reveals the earliest changes in microvascularization [44], PA-induced improvements in retinal microvasculature might reflect broader systemic vascular benefits in general. Such adaptations could promote more stable vessel architecture, improved perfusion, and reduced hypoxia, thereby potentially creating a less favorable microenvironment for tumor recurrence or progression [16,33,45].

Vascular remodeling of the TME is also considered one of the central mechanisms through which exercise may improve treatment response and, ultimately, survival in cancer patients [46]. The observation of PA-associated microvascular adaptations in close proximity to the ocular tumor may therefore represent a first indication that exercise-induced vascular normalization occurs not only systemically but also locally within the tumor-adjacent microenvironment. These local PA-induced vascular adaptations could have important therapeutic implications by reducing hypoxia, improving tissue perfusion, immune surveillance, and thus the effectiveness of tumor therapies. This warrants further investigation in prospective interventional designs. In this context, the ongoing STIMULATE trial (DRKS00035528) aims to examine whether microvascular adaptations induced by high-intensity interval training during acute chemotherapy differ between cancer patients and age-matched healthy controls.

In this context, preliminary evidence provides a promising basis for future investigations. Acute HIIT was shown to significantly alter retinal microvascular reactivity in young cancer patients undergoing chemotherapy, as assessed by OCTA [47]. Furthermore, a longitudinal case observation in a UM patient with metastatic disease under immunotherapy and following radiotherapy suggested that HIIT may contribute to the stabilization of OCTA-derived retinal parameters even in a progressive, non-curative oncological setting [48]. This underscores the potential of exercise-based interventions to preserve microvascular integrity across different stages and treatment contexts of ocular cancer.

The present findings suggests that these microvascular benefits are not restricted to high-intensity exercise regimens. Even moderate-intensity PA can induce meaningful adaptations at the microvascular level, as reflected in retinal vascular density parameters.

This is particularly relevant given that the majority of patients in the present study had already completed treatment, representing a post-therapeutic population in which long-term vascular health and tumor surveillance are of primary concern. Furthermore, given that retinal microvasculature is particularly vulnerable to treatment-related damage, such as radiation retinopathy, PA-induced improvements in vascular integrity may help attenuate or partially counteract these adverse effects. Taken together, our findings suggest that PA may represent a low-risk, accessible, and potentially beneficial supportive therapy for patients with UM, warranting further investigation in prospective and interventional study designs.

### Methodical Considerations

Several limitations of the present study warrant consideration. Notably, a lower image quality threshold was applied for tumor-affected eyes (QS ≥ 5) compared to healthy control eyes (QS ≥ 7), reflecting the greater technical difficulty of achieving high signal quality in UM eyes, in part due to the presence of the tumor within the imaged field. As OCTA-derived density metrics are known to correlate with signal strength, this asymmetry in quality thresholds cannot be fully excluded as a contributing factor to the observed reduction in vessel density parameters in UM eyes, independent of genuine tumor-related vascular remodeling. Similarly, axial length and refractive error, both factors that may influence OCTA-derived density measurements through magnification effects, were not assessed in the present study and cannot be excluded as an additional contributing factor to the observed group differences.

The time since diagnosis and treatment varied considerably across participants. Since the TME is not static but undergoes continuous vascular remodeling and structural adaptation following therapy [49], this heterogeneity may have introduced variability in the observed microvascular characteristics and should be more systematically addressed in future research. Detailed data on radiation dosage and fractionation were not systematically collected for the irradiated UM patients in the present study. As radiotherapy protocols can vary considerably in intensity and delivery, future studies should systematically document these parameters to enable a more precise characterization of radiotherapy-related microvascular changes.

Intraocular pressure and best-corrected visual acuity were recorded, yet their potential confounding effect on the observed group differences should be addressed in future research. Broader systemic risk profiling likewise remains an important avenue for future studies.

The relatively small and heterogeneous sample further limits statistical power, and future studies with larger, more homogeneous cohorts are warranted. Importantly, future research should prioritize the inclusion of treatment-naïve patients prior to radiotherapy initiation, enabling a clearer distinction between tumor-induced and radiation-induced microvascular alterations. Moreover, improved vascular parameters as assessed by OCTA should need to be directly linked to hard clinical endpoints, such as treatment response, recurrence rates, and overall survival, in order to establish the clinical significance of PA-associated microvascular adaptations in the TME. Furthermore, given the number of models tested, the reported associations involving PA carry a non-negligible risk of false-positive findings and should be interpreted with appropriate caution as exploratory rather than confirmatory.

Further, the choroidal vasculature was not included in the quantitative OCTA analysis, as reliable choroidal segmentation requires advanced correction algorithms beyond the scope of the present study. Moreover, choroidal perfusion is predominantly governed by systemic hemodynamics rather than local autoregulation, making sustained PA-related adaptations in this vascular bed less likely [41].

Finally, PA was assessed using the BSA-F 3.0 questionnaire, a self-report instrument well suited to the German-speaking context. However, susceptibility to recall and social desirability bias cannot be excluded. Furthermore, compared to internationally established assessments such as the IPAQ, the cross-cultural validation of the BSA-F remains more limited, which should be considered when interpreting and comparing the present findings with the international literature.

## 5. Conclusions

The present findings suggest that habitual PA is positively associated with retinal MVD in patients with uveal melanoma and ocular nevi, with higher PA levels corresponding to greater vascular density in the SVP. These associations were independent of diagnostic group, indicating that PA may exert beneficial microvascular effects regardless of the underlying ocular condition. Furthermore, significant differences in retinal microvascular parameters between UM and nevus patients were observed, consistent with tumor-induced vascular remodeling within the ocular TME.

In conclusion, the present study provides preliminary evidence that PA is associated with favorable retinal microvascular adaptations in UM patients and nevus. Future prospective and interventional studies with larger, well-characterized cohorts are needed to confirm these findings and to elucidate the underlying mechanisms.

## Figures and Tables

**Table 1:** Biometric characteristics and microvascular metrics. Abbreviations: BMI, body mass index; M, mean; *N*, number; MET, metabolic equivalent; SD, standard deviation.

	Nevus (*N* = 22)		Uveal Melanoma (*N* = 20)		*p*-Value
Sex, *N* (%)				Sex, *N* (%)	
f	16	(73)	9	(5)	
m	6	(27)	11	(55)	0.280 ^3^
Age, years, M (±SD)	56.14	(±13.48)	63.45	(±13.76)	**0.036 ^1^**
Height, m, M (±SD)	1.70	(±0.10)	1.71	(±0.10)	0.644 ^2^
Weight, kg, M (±SD)	84.08	(±21.03)	80.59	(±16.11)	0.617 ^1^
BMI (kg/m^2^), M (±SD)	27.42	(±10.07)	26.29	(±8.38)	0.677 ^2^
Best-corrected visual acuity (decimal scale), M (±SD)	0.86	(±0.14)	0.65	(±0.31)	**0.003**
Intraocular pressure, mmHG, M (±SD)	15.50	(±3.35)	12.70	(±3.40)	**0.011**
Charlson Comorbidity Index (CCI), M (±SD)	2.14	(±1.39)	2.10	(±1.37)	0.933 ^1^
Days diagnosis to assessment, M (±SD)	318.59	(±468.04)	796.80	(±754.14)	**0.006 ^2^**
Prior radiotherapy, *N* (%)	-	-	15	(75)	**-**
Days radiotherapy to assessment, M (±SD) ᵃ	-	-	686.67	(±619,76)	-
Enucleation during follow-up, *N* (%)	-	-	3	(15)	-
Tumor eye, *N* (%)					
right	9	(41)	15	(75)	
left	13	(59)	5	(25)	0.441 ^3^
Lesion size (Diameter), mm, M (±SD)	5.02	(±2.90)	9.05	(±3.65)	**0.010 ^2^**
Lesion thickness, mm, M (±SD)	0.68	(±0.85)	3.38	(±1.87)	**<0.001 ^2^**
MET-min/week M (±SD)	2855.91	(±2170.66)	2327.30	(±2482.91)	0.190 ^2^
**Superficial vascular plexus, M (±SD)**					
Nodes, *n*	1817.05	(±281.47)	1447.68	(±443.19)	**0.003 ^1^**
Vessel area density, %	36.67	(±1.92)	33.86	(±3.36)	**<0.001 ^1^**
Total vessel length, mm	394.48	(±26.26)	352.71	(±46.90)	**<0.001 ^1^**
Vessel length density_pct, %	7.89	(±0.53)	7.06	(±0.94)	**0.004 ^2^**
Vessel length density_mm, mm^−1^	15.78	(±1.05)	14.11	(±1.88)	**<0.001 ^1^**
Mean length, mm	100.80	(±4.37)	106.46	(±8.23)	**0.002 ^1^**
Mean diameter, mm	22.95	(±0.51)	22.96	(±0.58)	1.000 ^2^
Branchpoint density, nodes/mm^2^	4.58	(±0.47)	4.02	(±0.72)	**<0.001 ^1^**
Mean tortuosity	1.17	(±0.01)	1.17	(±0.01)	0.465
**Deep vascular plexus, M (±SD)**					
Nodes, *n*	3270.60	(±444.22)	2617.55	(±755.19)	**<0.001 ^1^**
Vessel area density, %	43.41	(±1.93)	40.05	(±4.11)	**<0.001 ^1^**
Total vessel length, mm	506.53	(±30.88)	453.46	(±66.75)	**<0.001 ^1^**
Vessel length density_pct, %	10.13	(±0.62)	9.07	(±1.34)	**<0.001 ^1^**
Vessel length density_mm, mm^−1^	20.26	(±1.24)	18.14	(±2.67)	**<0.001 ^1^**
Mean length, mm	82.70	(±4.19)	89.05	(±7.15)	**<0.001 ^1^**
Mean diameter, μm	22.30	(±0.47)	22.91	(±0.53)	**0.002 ^2^**
Branchpoint density, nodes/mm^2^	6.43	(±0.54)	5.67	(±0.89)	**<0.001 ^1^**
Mean tortuosity	1.19	(±0.01)	1.19	(±0.01)	0.366 **^1^**

*Note: bold numbers are indicating a significant difference (p < 0.05).* ^1^ *Student’s t-test;* ^2^ *Mann–Whitney U test; *^3^ *Fisher’s exact test; ᵃ Among UM patients with prior radiotherapy only (n = 15/20).*

**Table 2:** Summary of multiple linear regression analyses for superficial and deep macular OCTA parameters (*N* = 42). Abbreviations: UM, uveal melanoma; MET, metabolic equivalent. Age (at diagnosis) and the Charlson Comorbidity Index (CCI) were included in the initial regression models. As these variables did not reach statistical significance (*p* > 0.05) for any of the analyzed parameters, their corresponding coefficients are not displayed in this table for clarity.

	Group (UM vs. Nevus)					Physical Activity (log10 MET)			
	Model R^2^	Estimate (B)	Standard Error	*p*-value	η^2^	Estimate (B)	Standard Error	*p*-value	η^2^
**Superficial vascular plexus (SVP)**									
Nodes	0.48	−396.04	108.71	**<0.001**	0.113	94.54	63.59	0.146	0.147
Vessel area density	0.54	−3.05	0.76	**<0.001**	0.155	1.07	0.45	**0.022**	0.133
Total vessel length	0.56	−44.81	10.44	**<0.001**	0.159	13.98	6.11	**0.028**	0.120
Vessel length density_pct	0.56	−0.9	0.21	**<0.001**	0.158	0.28	0.12	**0.028**	0.119
Vessel length density_mm	0.56	−1.79	0.42	**<0.001**	0.159	0.56	0.24	**0.028**	0.120
Mean length	0.41	5.54	2.01	**0.009**	0.104	−2.21	1.17	0.068	0.103
Mean diameter	0.07	−0.11	0.21	0.61	0.000	−0.18	0.12	0.154	0.102
Branchpoint density	0.41	−0.6	0.19	**0.003**	0.090	0.11	0.11	0.305	0.165
Mean tortuosity	0.07	−0.001	0.003	0.654	0.033	0.002	0.00	0.201	0.018
**Deep vascular plexus (DVP)**									
Nodes	0.41	−687.82	196.28	**0.001**	0.158	187.36	114.81	0.111	0.061
Vessel area density	0.42	−3.58	1.01	**0.001**	0.186	1.05	0.59	0.084	0.043
Total vessel length	0.45	−54.51	15.82	**0.001**	0.164	15.04	9.25	0.113	0.046
Vessel length density_pct	0.45	−1.09	0.32	**0.001**	0.165	0.33	0.19	0.113	0.046
Vessel length density_mm	0.45	−2.18	0.63	**0.001**	0.164	0.62	0.37	0.113	0.046
Mean length	0.34	6.38	1.97	**0.003**	0.190	−1.96	1.15	0.097	0.030
Mean diameter	0.42	0.32	0.16	0.055	0.166	−0.07	0.09	0.442	0.014
Branchpoint density	0.33	−0.8	0.25	**0.002**	0.153	0.2	0.14	0.173	0.049
Mean tortuosity	0.07	0.01	0.00	0.192	0.002	−0.001	0.00	0.578	0.089

*Note: bold numbers are indicating a significant effect (p < 0.05).*

## Data Availability

The data presented in this study are available on reasonable request from the corresponding author.

## Supplementary Materials

The following supporting information can be downloaded at: https://www.mdpi.com/article/10.3390/cancers18152502/s1, Table S1: STROBE Statement—checklist of items that should be included in reports of observational studies.

## Abbreviations

The following abbreviations are used in this manuscript:

BD	Branchpoint density
BMI	Body Mass Index
BSA-F	Bewegungs- und Sportaktivität Fragebogen
CCI	Charlson Comorbidity Index
CIO	Centrum für Integrierte Onkologie
DRKS	Deutsches Register Klinischer Studien
DVP	Deep Vascular Plexus
ECMI	EyeCanMoveIt
eNOS	Endothelial Nitric Oxide Synthase
HIIT	High intensity interval training
IOP	Intraocular Pressure
IPAQ	International Physical Activity Questionnaire
M	Mean
MET-minutes/week	Metabolic Equivalent of Task in Minutes per Week
MVD	Microvascular Density
NO	Nitric Oxide
OCTA	Optical Coherence Tomography Angiography
OCTAVA	OCTA Vascular Analyzer
OD	Oculus Dexter
OLS	Ordinary Least Squares
OS	Oculus Sinister
PA	Physical Activity
QS	Quality Score
R^2^	Coefficient of determination
SD	Standard Deviation
SVP	Superficial Vascular Plexus
TME	Tumor Microenvironment
TSD	Time since Diagnosis
UM	Uveal Melanoma
VAD	Vessel Area Density
VLD_pct	Vessel Length Density (%)
VLD_mm	Vessel Length Density (mm^−1^)
